# Editorial: Addictions and eating behavior

**DOI:** 10.3389/fnut.2025.1584058

**Published:** 2025-03-21

**Authors:** Ramón Sotomayor-Zárate

**Affiliations:** Centro de Neurobiología y Fisiopatología Integrativa (CENFI), Instituto de Fisiología, Facultad de Ciencias, Universidad de Valparaíso, Valparaíso, Chile

**Keywords:** dopamine, food addiction, feeding, reward, eating behavior

Feeding is a complex process that involves multiple brain regions responsible for homeostasis, learning, memory, emotion, and reward (1, 2). Consequently, dysregulation of these areas can lead to hyperphagia, an increased preference for obesogenic foods, and, in the short or medium term, the development of overweight and obesity.

In this Research Topic of *Frontiers in Nutrition*, “*Addictions and Eating Behavior*”, we have published nine original articles authored by 59 researchers from Chile, China, Spain, Finland, Israel, Italy, and the United States, with more than 14,000 views and downloads. These articles provide novel insights into neurobiological mechanisms, genetic polymorphisms, and environmental factors that contribute to changes in eating behaviors, such as increased preference for obesogenic foods.

A primary focus of the published articles is the relationship between stress, anxiety, and eating behavior, ranging from appetite suppression to overeating. In this context, Peleg et al. demonstrate that emotional regulation, stress management, and the development of healthy coping mechanisms reduce the risk of binge eating, which otherwise leads to compulsive food consumption and, in the long term, increases the likelihood of developing overweight and obesity. The study by Marchena-Giráldez et al. highlights how emotional eating—the tendency to eat in response to both negative and positive emotions—is associated with excessive Internet use and the development of anxiety and stress-related traits, which may further promote overweight and obesity. Additionally, the work of Tan et al. identifies a positive correlation between social support and healthy eating behaviors in children and adolescents, independent of body mass index (BMI). This suggests that social support plays a crucial role in mitigating the risk of obesity and unhealthy eating behaviors.

A second key topic explored in this Research Topic concerns food addiction and its association with the compulsive consumption of high-fat, high-sugar foods, which activate the brain's reward system in a manner similar to drugs of abuse. In this context, Mastrobattista et al. investigate the relationship between food addiction and psychological risk factors such as impulsivity, social anxiety, and depressive disorder. Their findings suggest that public health initiatives should consider food addiction as a contributing factor to the development of chronic non-communicable diseases. Furthermore, Palacio et al. demonstrate that patients undergoing weight loss treatment exhibit a positive correlation between food addiction and higher body weight, waist circumference, and BMI. Friling et al. report that dietary supplementation with wild green oat extract reduces stress levels associated with smoking cessation, potentially mitigating the hyperphagia commonly observed during tobacco withdrawal.

A third key area covered in the accepted articles focuses on genetics, specifically how genetic variants influence obesity risk by affecting the regulation of hunger-satiety cycles, preference for obesogenic foods, and macronutrient metabolism. In this context, Dabin Yeum et al. show that the risk allele of the FTO gene (associated with fat mass and obesity) at locus 16q12.2 predisposes individuals to a heightened hedonic response to food, which is linked to dysfunction in homeostatic feeding regulation areas such as the lateral hypothalamus. The work of Luengo et al. provides evidence that genotypes associated with reduced dopaminergic signaling are linked to uncontrolled emotional eating, potentially leading to overweight and obesity. Finally, Sayers et al. demonstrate at a fundamental level that Pituitary Adenylate Cyclase-Activating Polypeptide (PACAP) neurons in the ventromedial hypothalamus can inhibit dopaminergic neurons in the ventral tegmental area, thereby reducing impulsive food intake. These findings offer new perspectives for the treatment of obesity and food addiction.

This Research Topic has significantly contributed to advancing scientific knowledge on the pathophysiological mechanisms underlying dysregulated eating behaviors and their role in the development of chronic non-communicable diseases associated with high BMI. We look forward to further exploring both pathophysiological and therapeutic aspects of addictions and eating behavior in future research endeavors.
